# Clinicopathological Analysis and Its Correlation With Various Classes of Lupus Nephritis

**DOI:** 10.7759/cureus.78065

**Published:** 2025-01-27

**Authors:** Nivedita J, Barathi Gunabooshanam, Subalakshmi Balasubramanian

**Affiliations:** 1 Pathology, Madras Medical College, Chennai, IND; 2 Pathology, Sri Ramachandra Institute of Higher Education and Research, Chennai, IND

**Keywords:** activity and chronicity indices, chronic kidney disease (ckd), class iv lupus nephritis, lupus nephritis, systemic lupus erythematosus

## Abstract

Background and objectives

Systemic lupus erythematosus (SLE) is a complex autoimmune disorder characterized by chronic immune complex deposition and involvement of multiple organ systems. Among individuals with SLE, a greater percentage are at a higher risk of developing lupus nephritis (LN). Renal biopsies play a pivotal role in diagnosing, managing, and predicting the prognosis of LN. The classification of LN relies heavily on light microscopy findings, supplemented by histochemistry, direct immunofluorescence, and electron microscopy. Lupus nephritis is categorized into six distinct classes based on the quantitative evaluation of histological lesions. The study aimed to analyze and correlate demographic data, biochemical parameters, serological values, and histopathological features in patients with LN who underwent renal biopsies to categorize the different classes of LN based on the WHO and International Society of Nephrology (ISN)/Renal Pathology Society (RPS) 2003 classification systems. The study also aimed to establish correlations with the National Institutes of Health (NIH) activity and chronicity indices of LN.

Methods and results

This retrospective study included 102 patients diagnosed with SLE who underwent renal biopsies confirming LN. Among the 102 cases of LN, patients were grouped into two categories: those with active LN (61 cases) and those without active LN (41 cases). The most commonly observed histological subtype was class IV/V LN, followed by isolated class IV LN, both of which were associated with higher activity scores based on the NIH scoring system for LN. The findings emphasize that the majority of patients with SLE present with features of active disease (class IV or combined class IV/V) at the time of diagnosis and often progress to chronicity within a short timeframe. Additionally, the presence of antibodies related to other autoimmune diseases also had an impact on patients' progression to end-stage renal disease (ESRD) and overall prognosis.

Conclusion

The study highlights the critical role of histological classification in diagnosing and predicting the prognosis of LN. Active LN, particularly class IV or class IV/V combined, is more common at diagnosis and often progresses to chronicity. Regular monitoring using the modified NIH activity and chronicity scores, along with routine renal profiling and urine analysis, is essential to support the need for repeat renal biopsies during treatment. This approach enables the early detection of higher-class transitions. The prognostic value of histological scoring has been well established, underscoring the importance of both activity and chronicity indices in guiding therapeutic strategies.

## Introduction

Systemic lupus erythematosus (SLE) is an autoimmune disease characterized by chronic immune complex formation with multiorgan involvement, predominantly affecting the kidneys. The incidence and prevalence rates range from 0.9 to 3.1 (per 100,000 per year) and 4.3 to 45.3 (per 100,000 per year) across Asia-Pacific countries, respectively [1]. Around 70% to 80% of SLE patients have a higher potential to develop lupus nephritis (LN) [2,3], and 10% of LN patients tend to progress to end-stage renal disease. The majority of these patients present with class IV diffuse proliferative glomerulonephritis and succumb to death or lose their renal function within five years after diagnosis. Renal biopsies play a significant role in the diagnosis, management, and, most importantly, the prognosis of patients with LN.

There is a strong correlation between the histomorphology changes in renal biopsies and clinical/laboratory findings. Histopathological findings determine the extent of parenchymal damage in the kidney, thus guiding the treatment and prognosis of patients with LN [4-13]. Lupus nephritis classification primarily depends on the light microscopic findings combined with histochemistry, direct immunofluorescence, and electron microscopy. The diagnosis also needs to be scored per the 2003 International Society of Nephrology (ISN)/Renal Pathology Society (RPS) classification into six different classes based on the quantitative assessment of the histological lesions [12].

## Materials and methods

Aims and objectives

The study aims to compare and correlate the demographic data, biochemical parameters, serological values, and histopathological findings in patients with LN. Furthermore, the study subclassifies LN in renal biopsies according to the WHO and the ISN/RPS 2003 classification [4] and correlates with the National Institutes of Health (NIH) activity and chronicity indices of LN.

Materials and methodology

This is a retrospective, single-center study of 102 patients with SLE who underwent renal biopsy at the Department of Pathology, Sri Ramachandra Medical College and Research Institute (Chennai, TN, IND) and were confirmed to have LN between January 2019 and July 2024. The study was conducted after obtaining Institutional Ethics Committee approval from Sri Ramachandra Medical College and Research Institute (approval no. CSP-MED/24/OCT/110/351). The patients fulfilling the revised American College of Rheumatology (ACR) criteria for SLE [6] as determined by their physicians had a renal biopsy to determine the class of LN. Renal biopsy-confirmed LN was classified according to the 2003 ISN/RPS LN classification, and the NIH activity and chronicity indices were given based on the glomerular morphologies [5,12].

Inclusion and exclusion criteria

Newly diagnosed adults with SLE and clinical suspicion for LN and those with known cases of LN undergoing treatment and testing to determine higher class switching were enrolled in the study. Patients who did not fulfill the ACR criteria for diagnosis of SLE, patients with any other primary diagnosis, transplanted kidneys, and primary neoplastic conditions were excluded from the study.

Data collection

Data were obtained from the medical records department, including demography and investigations. At the time of the renal biopsy, spot urine protein creatinine ratio (uPCR), 24-hour urine protein, complete blood count (CBC), renal function test (RFT), complement C3, C4, antinuclear antibody (ANA), serum double-stranded DNA (dsDNA), and anti-phospholipid antibodies were noted. Data about urine routine, uPCR, blood urea nitrogen (BUN), and serum creatinine were also collected. Proteinuria was defined as urine protein ≥1+ (30mg/dL) by dipstick test and spot uPCR >0.2 mg/dL. Patients with proteinuria of more than 500 mg/day were considered for a kidney biopsy. Microscopic hematuria was defined as >5 red blood cells/high power field, and active urinary sediments such as red blood cell casts were noted.

Histopathology data

Renal biopsies were performed under ultrasound guidance using an 18-gauge, 22 mm × 10 mm semi-automated renal biopsy gun. All renal biopsy specimens were examined using a light microscope and a direct immunofluorescence microscope. Hematoxylin and eosin (H&E), periodic acid Schiff (PAS), Jones methenamine silver (JMS), and Masson’s trichrome (MT) stains were performed for light microscopy as per the standard operating procedure. Specimens for immunofluorescence microscopy were stained using fluorescein isothiocyanate (FITC) conjugated polyclonal rabbit antisera against human IgG, IgM, IgA, C3c, C1q, kappa, lambda, fibrinogen, and albumin. Immunofluorescence findings were categorized based on location and intensity, from (+) to (+++).

The ISN/RPS 2003 criteria with NIH update was applied for scoring the activity and chronicity index in proliferative LN classes III, IV, and combined class III/V and IV/V [12]. Indicators of disease activity include endocapillary hypercellularity, neutrophils and karyorrhexis within the glomerular capillary loops, fibrinoid necrosis, hyaline deposits, cellular or fibro-cellular crescents, and interstitial inflammation. Indicators of disease chronicity include the total percentage of global glomerulosclerosis, fibrous crescents, tubular atrophy, and interstitial fibrosis. The scoring was given as 0 to 3, based on the percentage of glomeruli involved. Scores of 0 indicated no glomerular involvement, while scores of 1, 2, and 3 indicated 25%, 25% to 50%, and >50% glomerular involvement, respectively.

Statistical analysis

The data were analyzed, and the results were compared between LN cases with activity (classes III, IV, and combined IV+V/III+V) and those without activity (classes I, II, and V). The analysis is presented as numbers and percentages, and the significance of associations determined at a 95% confidence level with a p-value of less than 0.05 considered significant was calculated using the chi-square test. A box-and-whisker plot was used to illustrate the interquartile range for both activity and chronicity indices in LN classes III, IV, and combined IV+V/III+V.

## Results

We studied a total of 102 cases of LN and divided them into two broad categories: LN with activity (61) and LN without any activity (41). Three cases of class I LN, 17 cases of class II LN, 11 cases of class III LN, 21 cases of class IV LN, 21 cases of class V LN, five cases of class III + V LN, and 24 cases of class IV + V LN were identified. The most frequently found histological type was class IV + V LN, followed by combined class IV.

The study population included patients in the age groups ranging from 16 to 55 years with a mean age of 40 years. Of which, 92 were female and 10 were male. Mean serum C3 and C4 values were typically low in active LN. Anti-nuclear antibody was positive in 99 cases and negative in three cases. The dsDNA levels were found to be high (>30 IU/mL) in 93 cases and normal in nine cases. Of note, seven cases were also antiphospholipid antibody (APLA) positive, five cases were positive for rheumatoid factor, three cases showed U1-RNP antibody in association with mixed connective tissue disorder, five cases were DCT positive without features of hemolysis, two cases were associated with small vessel vasculitis, seven cases were associated with hypothyroidism, and there was a single case with anti-TPO antibody positivity.

Glomerular compartment

We compared the following activity and chronicity parameters among the obtained LN classes according to the modified NIH activity and chronicity index (score 4), endocapillary hypercellularity, cellular crescent, fibrous crescent, neutrophilic infiltration, fibrinoid necrosis, segmental sclerosis, and global glomerulosclerosis. Endocapillary hypercellularity was noted in 53 cases (51.9%, p = 0.575), and neutrophilic infiltration was observed in 57 cases (55.8%, p = 0.001). The main indicating parameters of active disease, endocapillary proliferation, and karyorrhexis were found to be high in incidence in the overall 102 cases, highlighting the activity of the disease. Cellular crescent was present in 29 cases (28.4%, p = 0.001) and fibrinoid necrosis in 21 cases (20.5%, p = 0.001). Twenty-four cases (23.5%, p = 0.001) showed diffuse global glomerulosclerosis, 38 cases (37.3%, p = 0.024) showed interstitial inflammation, 37 cases (36.3%, p = 0.001) had tubular atrophy, and interstitial fibrosis was observed in 35 cases (34.3%, p = 0.001), which was statistically significant. Table 1 compares the parameters of the activity and chronicity score. Table 2 highlights activity parameters with a higher incidence in classes III and IV and combined IV+V/III+V.

**Table 1:** Histopathological data of LN with active and non-active LN The p-values for the parameters of the NIH activity and chronicity indices were calculated using the chi-square test. LN: Lupus nephritis, NIH: National Institutes of Health

Histopathological finding	LN with active disease (n = 61)	LN without active disease (n = 41)	p-value
Endocapillary proliferation	53 (51.9%)	49 (48%)	0.575
Karryorhexis	57 (55.8%)	45 (44.1%)	0.001
Hyaline deposits	47 (46.1%)	45 (44.1%)	0.778
Fibrinoid necrosis	21 (20.5%)	81 (79.4%)	0.001
Cellular or fibrocellular crescents	29 (28.4%)	63 (61.7%)	0.001
Interstitial inflammation	38 (37.3%)	54 (52.9%)	0.024
Global sclerosis	24 (23.5%)	68 (66.6%)	0.001
Tubular atrophy	37 (36.3%)	55 (53.9%)	0.001
Interstitial fibrosis	35 (34.3%)	57 (55.9%)	0.001
Fibrous crescents	2 (1.9%)	100 (98%)	0.001

**Table 2:** Comparison of NIH activity and chronicity index among proliferative classes of LN LN: Lupus nephritis, NIH: National Institutes of Health

LN with activity	Endocapillary proliferation	Karyorrhexis	Fibrinoid necrosis	Hyaline deposits	Cellular/fibrocellular crescents	Interstitial inflammation	Global sclerosis	Fibrous crescent	Tubular atrophy	Interstitial fibrosis
Class III n=11	9	8	3	4	5	7	4	0	6	6
Class IV n=21	21	21	9	21	15	14	8	2	9	9
Class III/V n=5	5	4	1	2	0	0	2	5	4	0
Class IV/V n=24	23	24	8	20	9	17	10	17	17	0

Tubulointerstitial and vascular compartment

We compared interstitial inflammation and tubular atrophy/interstitial fibrosis (IFTA) among the obtained classes of LN. Interstitial inflammation and IFTA were graded as follows. None in 25 cases, mild (<25% involvement) in 33 cases, moderate (25% to 50% involvement) in eight cases, and severe (>50% involvement) in one case.

Arterial changes like hyalinosis and medial wall hypertrophy were found in 54 cases. Of which, 51 cases were found to be mild in degree. Three cases showed severe arterial sclerosis with marked luminal narrowing.

**Figure 1:**
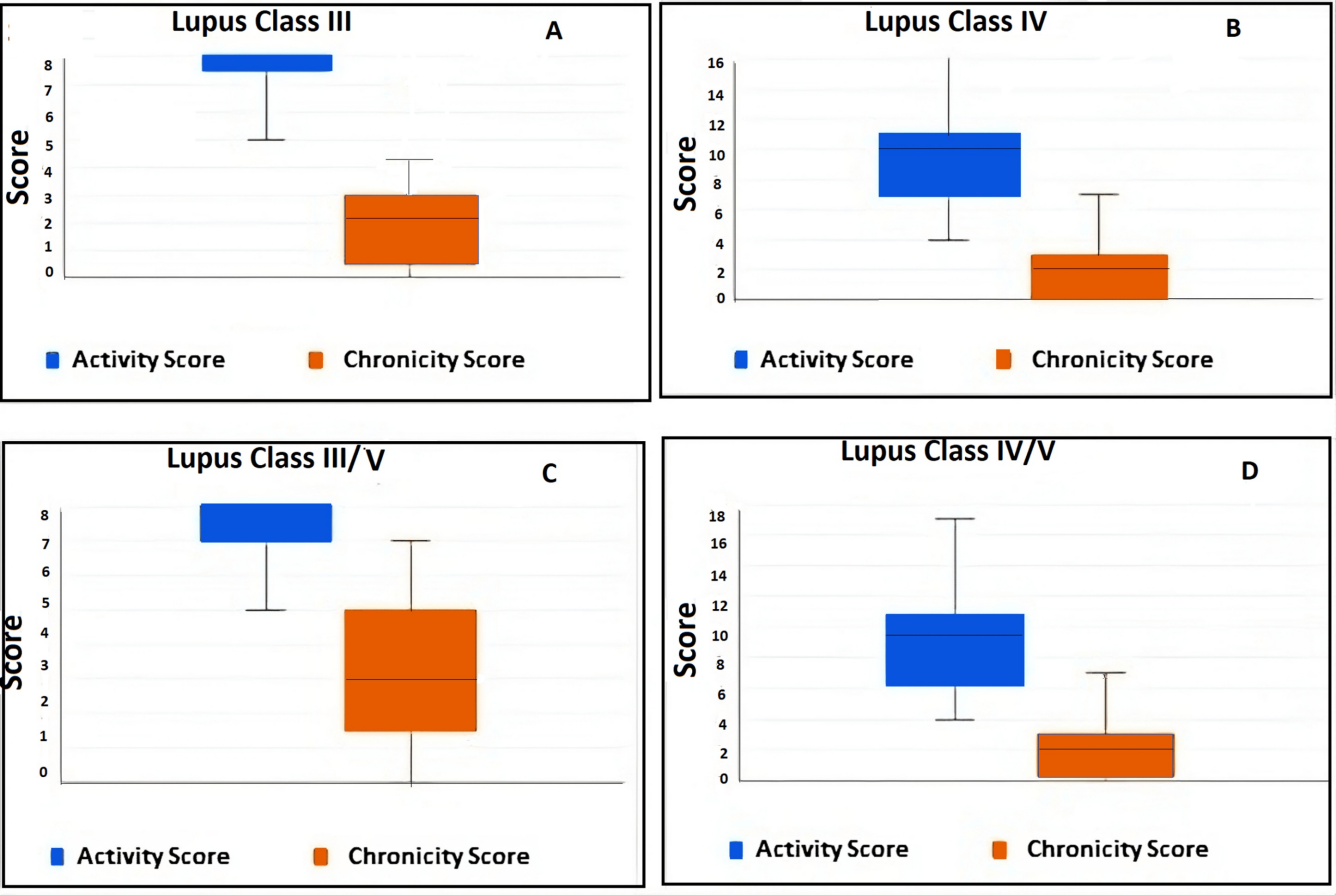
Box and whisker plot showing interquartile range of activity and chronicity score A: The activity score of lupus class III shows a wider interquartile range (IQR) compared to the chronicity score, demonstrating that active inflammation is more pronounced than long-term damage in this class. B: The activity score of lupus class IV) with a large IQR reflects substantial variability in disease activity. The chronicity score has a smaller IQR and lower median, suggesting that chronic damage is less prominent compared to active inflammation in class IV. C: The activity score for combined class III/V has a higher median score and variability compared to the chronicity score. The chronicity score with a narrower distribution represents that the chronic damage is relatively uniform and less significant in this class. D: The activity score lupus combined IV/V has the highest median and widest IQR among all classes, indicating significant and variable active disease. The chronicity score remains lower, with less variability, underscoring the predominance of active inflammation over chronic damage in this combined class.

**Figure 2:**
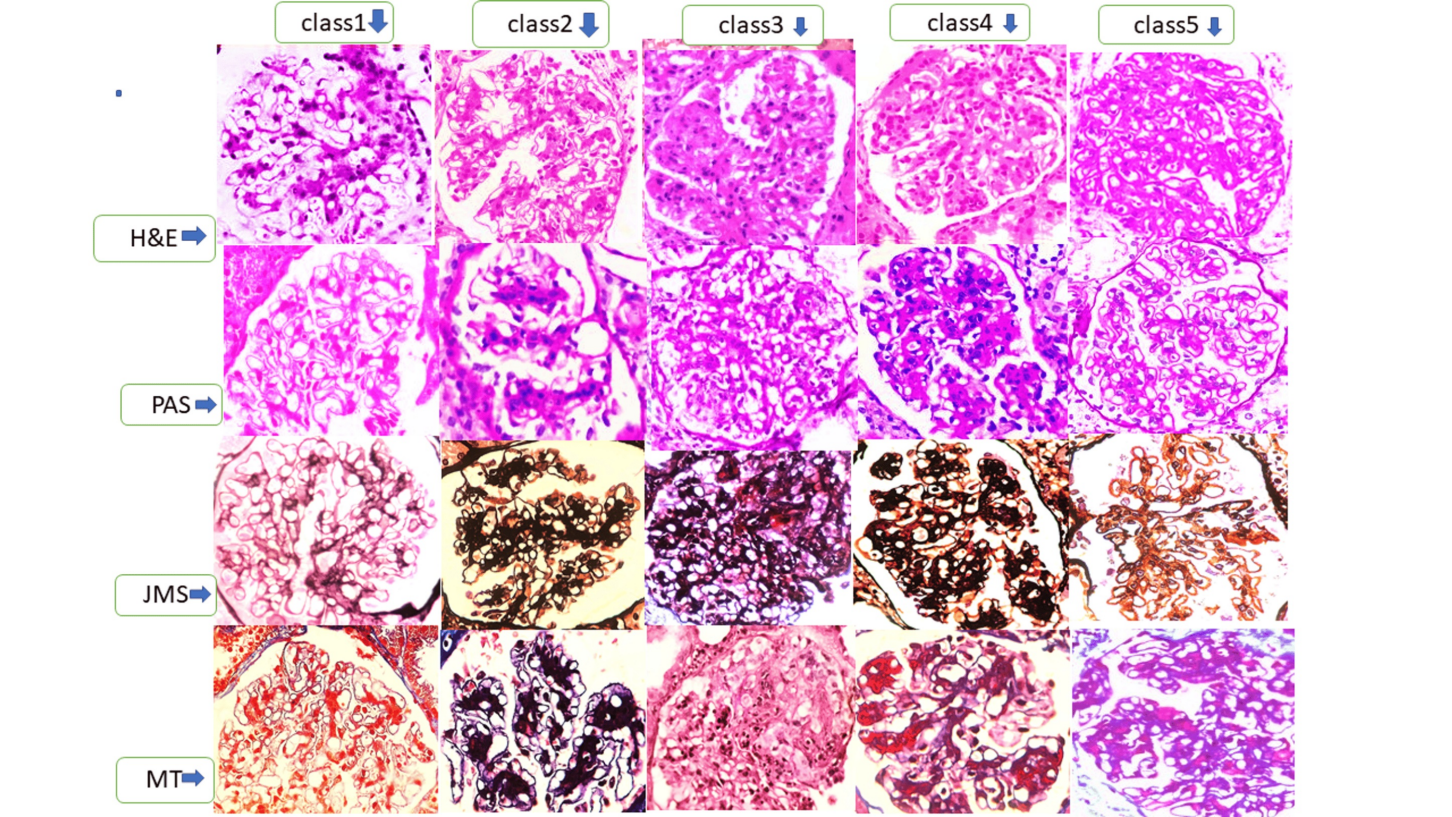
A collage of various classes of LN LN: Lupus nephritis, H&E: Hematoxylin and eosin, PAS: Periodic acid Schiff, JMS: Jones methenamine silver, MT: Masson trichrome Class I: Minimal mesangial LN showing normal glomerulus with no or minimal mesangial expansion; Class II: Mesangial proliferative LN showing mesangial hypercellularity appreciated in H&E and JMS; Class III: Focal LN showing segmental endocapillary proliferation and was noted in <50% of all glomeruli; Class IV: Diffuse LN showing endocapillary and mesangial hypercellularity, subendothelial hyaline deposits in JMS, and fibrinoid necrosis in MT; Class V: Thickened capillary wall basement membrane of LN (×400)

**Figure 3:**
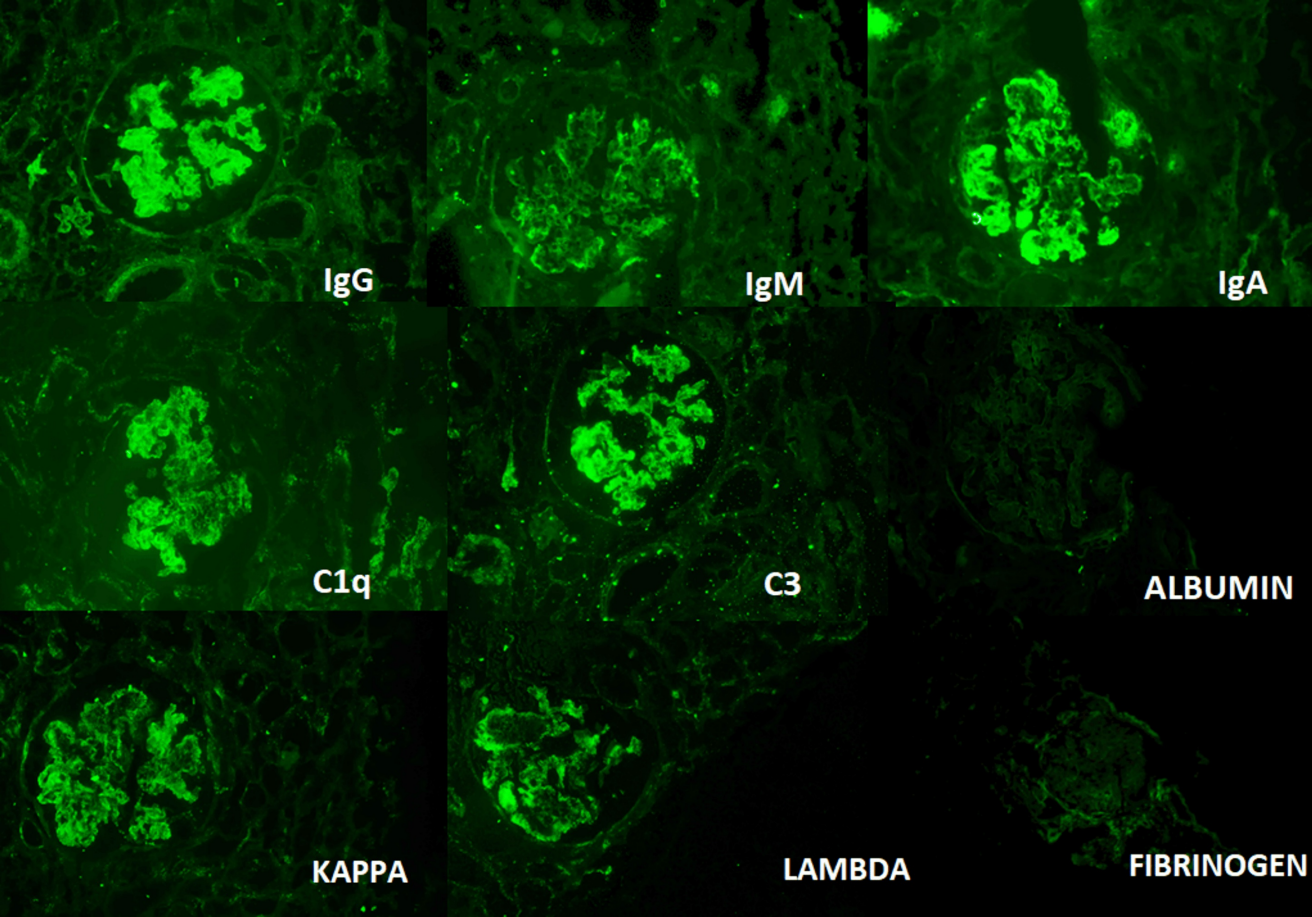
Direct Immunofluorescence from a class IV LN showing full house positivity Diffuse granular capillary wall and mesangial 3+ to 4+ positivity for immunoglobulins (IgG, IgM, IgA); complement (C1q and C3), kappa and lambda light chains noted. Fibrinogen and albumin are negative (×100). LN: Lupus nephritis

The box and whisker plots (Figure 1) for the active disease, class III, IV, combined III/IV, and IV/V showed that activity is higher than chronicity, and LN is proliferative and active during presentation. Figure 2 shows the H&E, PAS, JMS, and MT stains (x400) of class I to V LN. Figure 3 represents the full house positivity for all conjugates (IgG, IgM, IgA, C1q, C3, kappa, lambda) on direct immunofluorescence in class IV LN.

## Discussion

Renal involvement in SLE is common and often determines the course of the disease. The glomerular lesions that frequently accompany SLE have been the subject of intense investigation by clinicians and pathologists. The recent ISN/RPS 2003 classification aims to enhance the quality of communication among renal pathologists and clinical nephrologists regarding pathologic findings in lupus nephritis. The incidence of presentation in females was higher with a greater percentage: 88 cases in age < 40 years and 12 cases in females > 40 years of age. Ten cases were reported in male patients. We observed a predominance of proliferative glomerulonephritis (GN) over non‑proliferative LN in both genders.

Analysis performed to assess the relationship between clinical parameters and renal pathological activity showed a direct correlation of uPCR, microscopic hematuria, and anti-dsDNA antibody levels positively correlating with the renal pathological activity index. The activity scores were much higher in all cases of LN III/IV and combined III+V/IV+V. However, dsDNA was found to be negative in nine cases, and ANA was negative in three cases.

A positive correlation was observed between elevated BUN and serum creatinine with the presence of chronic lesions like glomerulosclerosis and tubulointerstitial chronicity (high chronicity index) on renal biopsy. The presence of a high degree of interstitial fibrosis in the first renal biopsy is associated with the development of end-stage renal disease (ESRD). The combination of a cellular crescent and interstitial chronicity is particularly ominous.

Three subsets of SLE patients were positive for U1-RNP antibody with features of connective tissue disorder. Mixed connective tissue disease (MCTD), regarded as a distinct autoimmune condition, can also serve as a precursor to other autoimmune diseases, such as SLE. Cases with dual-positive anti-SM and anti-RNP antibodies tended to show an early onset, with membranous nephropathy being the most prevalent pathological feature [14]. Differentiating between MCTD and SLE is critical, particularly because anti-RNP antibodies, a hallmark diagnostic criterion for MCTD, are also frequently observed in patients with SLE. Also, five cases tested positive for rheumatoid factor and one case positive for Sjogren’s SS-A/SS-B antibody. Seven cases of SLE showed features of hypothyroidism, with one being positive for anti-TPO antibody. According to Antonelli et al. [15], hypothyroidism is the most prevalent thyroid disorder among patients with lupus, with primary hypothyroidism affecting 15% to 19% of this population [15]. The prevalence is particularly pronounced in patients under 20 years of age. Female SLE patients show a higher propensity for both clinical and subclinical hypothyroidism compared to their male counterparts. In the current study, all four hypothyroid cases were females. The presence of thyroid dysfunction is linked to prolonged SLE disease activity. Systemic lupus erythematosus has been identified to hold the distinction of an autoimmune disease with over 180 diverse arrays of detectable antibodies [15,16].

Antiphospholipid antibody was positive in seven cases of SLE. These antibodies, initially described in lupus patients, are detected in 30% to 40% of SLE cases, though only 10% to 15% of SLE patients develop clinical antiphospholipid syndrome (APS). The presence of APLAs is an independent predictor of renal function decline in lupus nephritis. Among lupus patients with APLA positivity undergoing renal biopsy, the incidence of APS nephropathy can reach up to 40%, either as a standalone condition or coexisting with LN. Renal biopsy is indispensable for diagnosing APS nephropathy, as it is clinically and diagnostically challenging to distinguish from LN through laboratory investigations alone. Numerous studies have explored the role of angiogenic and anti-angiogenic factors in differentiating LN, APS nephropathy, and pre-eclampsia, but no definitive conclusions have been reached.

Five cases of LN were positive for the direct Coombs test (DCT), and two cases were in association with leukocytoclastic vasculitis. The direct antiglobulin test (DAT) is usually positive in autoimmune hemolytic anemia (AIHA). Hanaoke et al. reported cases of positive DCT in the absence of hemolytic anemia. This shows a higher predilection of lupus disease activity and poor renal response in LN. Direct antiglobulin test-negative hemolytic anemia has been reported in approximately 5% to 10% of patients [17]. The association of diverse autoantibodies in cases of LN could be a provoking cause of the highest incidence of active LN and progression to higher class switching and ESRD.

The limitation of this study is that it is retrospective in design. The possibility of a multicenter collaboration with data on the association with a diverse array of autoantibodies and long-term follow-up could provide a broader perspective on understanding the prognosis in active LN.

## Conclusions

The study highlights that most of the patients with SLE present with the features of active disease (class IV/combined class IV/V) at the time of diagnosis and move on to chronicity in a short course. The modified NIH activity and chronicity scores with routine renal profiles and urine examinations are necessary to substantiate a repeat renal biopsy during therapy to identify patients with higher class switching at an early stage. Also, serological examination for other antibodies by indirect immunofluorescence and confirmation by enzyme-linked immunosorbent assay (ELISA) is of great importance in patients with SLE, as the presence of other antibodies related to a few other autoimmune diseases also has an impact on the patient's progression to ESRD and prognosis. Certain antibodies, such as APLA, independently predict renal function deterioration. We emphasize the critical role of light microscopy and direct immunofluorescence studies in assessing the extent of renal damage through activity and chronicity indices, which serve as pivotal factors in guiding the treatment strategy for LN. The prognostic significance of histological scoring has been extensively evaluated, underscoring that both activity and chronicity indices are instrumental in shaping effective therapeutic interventions.
